# A Robust and Highly Precise Alternative against the Proliferation of Intestinal Carcinoma and Human Hepatocellular Carcinoma Cells Based on Lanthanum Strontium Manganite Nanoparticles

**DOI:** 10.3390/ma14174979

**Published:** 2021-08-31

**Authors:** Ali Omar Turky, Miral A. Abdelmoaz, Mahmoud M. Hessien, Ali M. Hassan, Mikhael Bechelany, Emad M. Ewais, Mohamed M. Rashad

**Affiliations:** 1 Central Metallurgical Research and Development Institute, P.O. Box 87, Helwan 11912, Cairo, Egypt; drewais65@gmail.com (E.M.E.); rashad133@yahoo.com (M.M.R.); 2 Chemistry Department, Faculty of Science, Al Azhar University, Nasar City 11765, Cairo, Egypt; alimhassanuk@yahoo.uk.com; 3 Institut Européen des Membranes, IEM, UMR 5635, University Montpellier, CNRS, ENSCM, 34070 Montpellier, France; mikhael.bechelany@umontpellier.fr; 4 Pharmaceutical Chemistry, Faculty Pharmacy, Sinai University, Arish 41611, Kantara, Egypt; miralahmed74@gmail.com; 5 Department of Chemistry, College of Science, Taif University, P.O. Box 11099, Taif 21974, Saudi Arabia; hessienmahmoud@yahoo.com; 6 Academy of Scientific Research and Technology, Cairo 11516, Cairo, Egypt

**Keywords:** lanthanum strontium manganite, nanoparticles, characterization, anticancer, antimicrobial

## Abstract

In this report, lanthanum strontium manganite at different Sr^2+^ ion concentrations, as well as Gd^3+^ or Sm^3+^ ion substituted La_0.5−*Y*_M*_Y_*Sr_0.5_MnO_3_ (M = Gd and Sm, *y* = 0.2), have been purposefully tailored using a sol gel auto-combustion approach. XRD profiles confirmed the formation of a monoclinic perovskite phase. FE-SEM analysis displayed a spherical-like structure of the La_0.8_Sr_0.2_MnO_3_ and La_0.3_Gd_0.2_Sr_0.2_MnO_3_ samples. The particle size of the LSM samples was found to decrease with increased Sr^2+^ ion concentration. For the first time, different LSM concentrations were inspected for their cytotoxic activity against CACO-2 (intestinal carcinoma cells) and HepG-2 (human hepatocellular carcinoma cells). The cell viability for CACO-2 and HepG-2 was assayed and seen to decrease depending on the Sr^2+^ ion concentration. Half maximal inhibitory concentration IC_50_ of CACO-2 cell and HepG-2 cell inhibition was connected with Sr^2+^ ion ratio. Low IC_50_ was noticable at low Sr^2+^ ion content. Such results were correlated to the particle size and the morphology. Indeed, the IC_50_ of CACO-2 cell inhibition by LSM at a strontium content of 0.2 was 5.63 ± 0.42 µg/mL, and the value increased with increased Sr^2+^ ion concentration by up to 0.8 to be = 25 ± 2.7 µg/mL. Meanwhile, the IC_50_ of HepG-2 cell inhibition by LSM at a strontium content of 0.2 was 6.73 ± 0.4 µg/mL, and the value increased with increased Sr^2+^ ion concentration by up to 0.8 to be 31± 3.1 µg/mL. All LSM samples at different conditions were tested as antimicrobial agents towards fungi, Gram positive bacteria, and Gram negative bacteria. For instance, all LSM samples were found to be active towards Gram negative bacteria Escherichia coli, whereas some samples have presumed antimicrobial effect towards Gram negative bacteria Proteus vulgaris. Such results confirmed that LSM samples possessed cytotoxicity against CACO-2 and HepG-2 cells, and they could be considered to play a substantial role in pharmaceutical and therapeutic applications.

## 1. Introduction 

Cancer is a disease of complex pathogenesis where part of the body grows and reproduces uncontrollably, with the prospect to infest or extend to other parts of the body [1,2,3,4,5]. Accordingly, World Health Organization (WHO) reports state that the primary and secondary reasons for death in humans before the age of 70 in 91 countries is cancer [6]. In this regard, nanotechnology has been predicted to revolutionize cancer management by the early detection of cancer in vivo, its rapid molecular analysis ex vivo, and subsequent anti-cancer therapy. Nanoparticles can negatively or efficaciously target tumors according to transfer contrast agents, as well as their size and type of therapy [6]. The nanoparticle size (a) endorses them to permeate even small blood vessels and (b) augments their negative uptake in tumor cells. Negative targeting utilizes streaming and porous tumor vasculature to permit nanoparticles and macromolecules to accumulate in interstitial spaces [7,8]. Meanwhile, minimized lymphatic evacuation from tissue assists in keeping particles coagulated in tumors [9,10]. Efficaciously targeting is accomplished by joining the targeting molecules with the particle, creating nanoparticles with targeting molecules bearing affinity towards antigens or receptors on tumor cells. In this context, magnetic nanoparticle MNPs are the ultimate reconnoitered particle models in medicine. Hyperthermia, bio-sensing, drug delivery, bio-separation, magnetic resonance imaging, and bio-separation are the common potential applications of magnetic nanoparticles. In regards to this, MNPs have been used considerably in the improvement of the magnetic separation of cancer cells, as contrast agents in tumor visualization, as anti-cancer theragnostics [11,12,13], and as disclosure marks in the diagnosis of cancer biomarkers. [14,15,16]. 

Lanthanum-strontium manganite (LSM) nanoparticles belongs to the distorted perovskite structure ABO_3_ and have a wide range of applications in medicine, fuel cells, electronics, solar cells, catalysis, and so on [17,18]. These materials have previously been tested for the hyperthermia treatment of cancer [19,20,21]. Herein, the formation of Lanthanum strontium manganite, La_1−x_Sr_x_MnO_3_ (LSM, *x* = 0.2, 0.5 and 0.8), as well as Gd^3+^ or Sm^3+^ ion substituted, La_0.5−*Y*_M*_Y_*Sr_0.5_MnO_3_ (M = Gd and Sm, *y* = 0.2), has been successfully fabricated using a sol gel auto-combustion approach based on citric acid as a fuel and a complexing agent. The prepared materials are examined as a chemotherapy towards CACO-2 (intestinal carcinoma) and HepG-2 (hepatocellular carcinoma cells). The selection of CACO-2 (intestinal carcinoma) is due to a renowned ferocious tumor of the digestive tract, which is the second most prevalent gastrointestinal tumor [22]. Furthermore, hepatocellular carcinoma cells (HepG-2 or HCC) are the most common (70–90%) among primary liver cancers worldwide. Finally, all samples were tested as antimicrobial agents towards fungi, Gram positive bacteria, and Gram negative bacteria.

## 2. Materials and Methods

### 2.1. Materials

As mentioned in our previous publication [23,24], different pure chemicals were utilized to tailor lanthanum strontium manganite (LSM) nanopowders based on an organic acid precursor strategy at different Sr^2+^ ion concentrations, as well as being substituted by Sm^3+^ and Gd^3+^ ions. Furthermore, bi-distilled water was employed in the present study.

### 2.2. Procedure

LSM nanomaterials have been purposefully developed based on a citrate precursor strategy. The procedure for fabrication of La_1−*x*_Sr*_x_*MnO_3_ at various Sr^2+^ ion content has been described in details in a previously study by our group [23]. Gd^3+^ ion substituted LSM nanopowders at a Gd^3+^ ion ratio of 0.2 has also been mentioned in other work [24]. Sm^3+^ ion replaced LSM nanopowders were also processed with similar trends of Gd content.

### 2.3. Physical Characterization

Phase evolution and crystallite size were realized based on XRD using a model Bruker AXS diffractometer D8-ADVANCE. The morphology of the produced nanopowders was accomplished by employing FE-SEM microscopy (JEOL-JSM-5410). 

### 2.4. Procedure and Materials for CACO-2 and HepG-2 Cells Treatment

The materials and cell line propagation of CACO-2 and HepG-2 cells have previously been explained in detail [25,26].

## 3. Results and Discussion 

### 3.1. Crystal Structure

**Figure 1:**
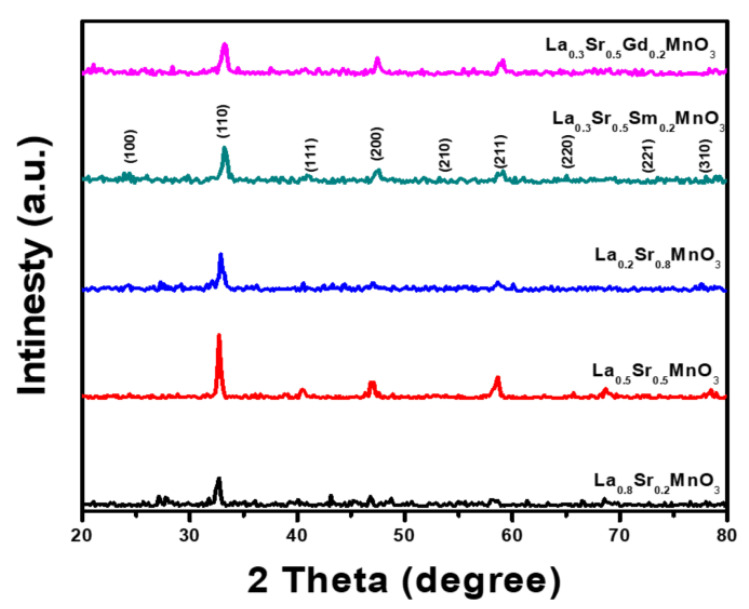
XRD patterns of lanthanum strontium manganite nanoparticles synthesized using a citrate precursor approach annealed at 1000 °C for 2h for different samples: LSM 0.2 Sr^2+^, LSM5 0.5 Sr^2+^, LSM8 0.8 Sr^2+^, LSMSm 0.2 Sm^3+^, and LSMGd 0.2 Gd^3+^.

Figure 1 shows the XRD profiles of lanthanum strontium manganit, La_1−*x*_Sr*_x_*MnO_3_ (LSMO), tailored using a sol gel auto-combustion approach with various S^2+^ ion molar ratios (*x* = 0.2, 0.5 and 0.8) as well as La_0.3_M_0.2_Sr_0.5_MnO_3_ (M = Sm^3+^ and Gd^3+^ ions), annealed at 1000 °C for 2 h. The assignment of the main peaks is assumed to be linked with different crystalline planes of LSMO and are in good correlation with reference card numbers (00-056-0616) and (89–4466) for LSMO (0.27) and LSMO (0.33), respectively [27]. Most main peaks located at 2θ = 32.90° and 2θ = 32.80° for La_1__−_*_x_*Sr*_x_*MnO_3_ at different Sr^2+^content have 25.2, 28.8, and 36.4 nm, respectively. Meanwhile, the crystallite size of the Gd^3+^ or Sm^3+^ substituted La_0.3_M_0.2_Sr_0.5_MnO_3_ was 32.6 and 35.9 nm, respectively.

### 3.2. Morphological Structure

**Figure 2:**
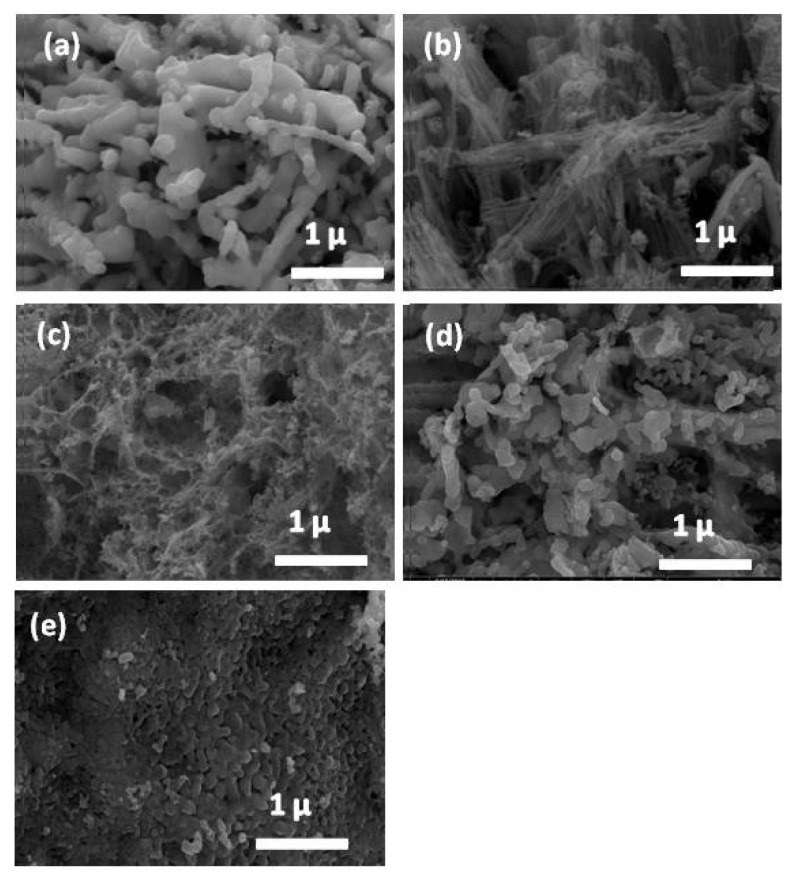
FE-SEM micrographs of (**a**) sample LSM2; LSM at 0.2 Sr^2+^ ion, (**b**) sample LSM5; LSM at 0.5 Sr^2+^ ion, (**c**) sample LSM8; LSM at 0.8 Sr^2+^ ion, (**d**) sample LSM Sm; La_0.3_Sm_0.2_Sr_0.5_MnO_3_, (**e**) sample LSM Gd; La_0.3_Gd_0.2_Sr_0.5_MnO_3_, synthesized through a citrate precursor pathway annealed at 1000 °C for 2 h.

The detailed microstructures of the LSMO samples at various synthesis conditions were inspected by FE-SEM, and the corresponding images for all samples are shown in Figure 2. As seen in Figure 2a, for the sample Sr^2+^ ion concentration of 0.2, the sample consists of agglomerated nanoparticles, and most of the grains are spherical with a cluster-like shape. However, Figure 2b, representing Sr^2+^ ion content of 0.5, shows that the agglomerated nanoparticles are stacked together to form a stick-like shape. Finally, Figure 2c, detailing La_0.8_Sr_0.2_MnO_3_, indicates that the agglomerated nanoparticles are connected with each other in a homogenous shape to form a spider-web-like structure. Furthermore, the Gd^3+^ion substituted and Sm^3+^ ion substituted LSM samples exhibited a spherical-cluster structure and one with different shapes, respectively. The grain size in the samples is distributed in the range of 20–50 nm.

### 3.3. Cytotoxicity Study against CACO-2

**Figure 3:**
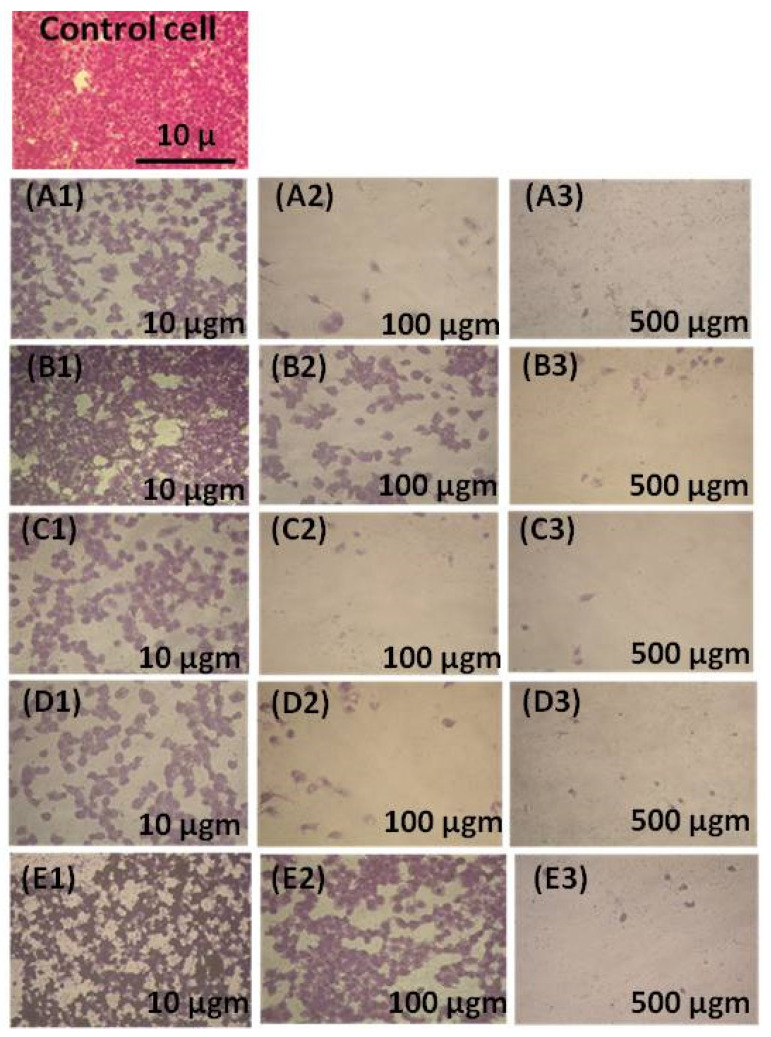
SEM images for CACO-2 control untreated sample (X) and samples treated with 10 µgm (**A1**,**B1**,**C1**,**D1**,**E1**), 100 µgm (**A2**,**B2**,**C2**,**D2**,**E2**), and 500 µgm (**A3**,**B3**,**C3**,**D3**,**E3**).

**Figure 4:**
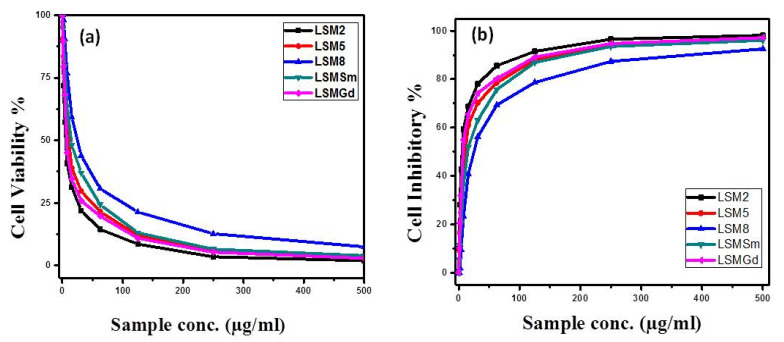
(**a**) The cell viability and (**b**) cell inhibitory (%) versus concentration of LSM with different Sr^2+^ ion content and LSM with 0.1 Sm ions and 0.1 Gd ions at concentrations from 0 to 500 µg/m against CACO-2 cell line.

**Figure 5:**
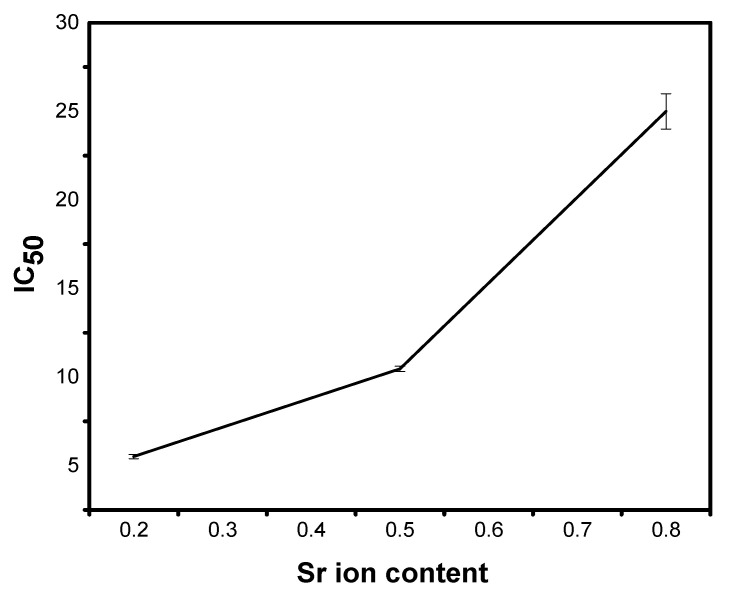
The relation between IC_50_ and Sr^2+^ ion content of lanthanum strontium manganite prepared by citrate precursor method.

Figure 3 presents the SEM images for the CACO-2 untreated control sample, X, and samples treated with 10 µgm: A1, LSM2; B1, LSM5; C1, LSM8; D1, LSMSm; and E1, LSMGd as well as with 100 µgm (A2, B2, C2, D2, and E2), and 500 µgm (A3, B3, C3, D3, and E3). Plainly, the cell viability was decreased and cell inhibition was increased by increasing the concentration of LSM samples at different conditions, as can be seen in Figure 4. Cell inhibition was found to be 90% with the addition of 500 µgm LSM2 and LSMGd. Half the maximal inhibitory IC_50_ concentration, in which 50% of the carcinoma was inhibited, was recorded. Low IC_50_ indicates high cytotoxicity, and high IC_50_ indicates low cytotoxicity. It is clear that the LSM8 sample at a high Sr^2+^ ratio had IC_50_ = 25 ± 2.7 µg/mL, and the LSM5 sample had IC_50_ at 10.6 ± 2.1, whereas the LSM2 sample had 5.63 ± 0.42 µg/mL. Consequently, half maximal inhibitory concentration IC_50_ was found to increase with increased Sr^2+^ ion content, as illustrated in Figure 5. Therefore, the inhibition toward the CACO-2 cell line was decreased with increased Sr^2+^ ion content. Meanwhile, the IC_50_ for the LSMGd sample was found to be 6.79 ± 0.36 µg/mL, whereas LSMSm was found to be 14.6 ± 1.9 µg/mL. Consequently, the optimum samples for inhibition of CACO-2 intestinal carcinoma were LSM2 and LSMGd. These samples have high inhibitions towards the tumor of CACO-2 cells compared with Cu-Nanoparticles, in which IC_50_ was 11.21 μg/mL [28]. Interestingly, they possessed a remarkable non-cytotoxic effect compared with Schiff based ligands and its two M (II) complexes, [CoCl·L(H_2_O)_2_]·2H_2_O, [RuCl (*p*-cymene) L], which were found to be promising anticancer agents [29]. The results can be attributed to the decreasing of the crystallite size of LSM2 and LSMGd compared with different samples. Furthermore, the surface area of the particles was decreased with decreasing particle size, which leads to simplifying the diffusion of particles into cells. In this context, The NPs can inhibit the cell viability by various mechanisms, including apoptosis and necrosis. Apoptosis is a cell suicide mechanism that commands cell numbers. The apoptosis mechanism is a composition of programmed cell death that results in the orderly and efficacious removal of damaged cells using an anticancer compound. Accordingly, LSM nanoparticles combine with chemical species in the tumor cell and create reactive oxygen species, leading to oxidative stress. This, in turn, leads to DNA damage, protein denaturation, and lipid peroxidation, which is mostly produced in cell death by apoptosis [30,31]

### 3.4. Cytotoxicity Study against HepG-2

**Figure 6:**
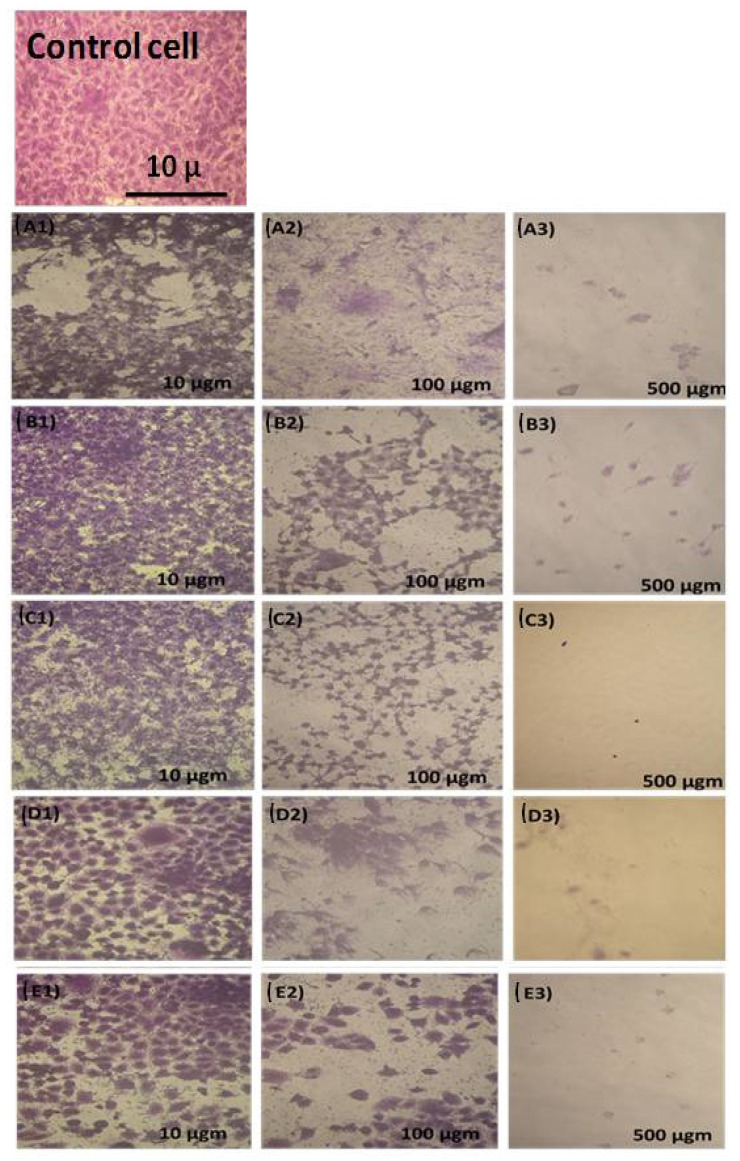
SEM images for HepG-2 cell control untreated sample and treated samples by 10 µgm (**A1**,**B1**,**C1**,**D1**,**E1**),100 µgm (**A2**,**B2**,**C2**,**D2**,**E2**), and 500 µgm (**A3**,**B3**,**C3**,**D3**,**E3**).

**Figure 7:**
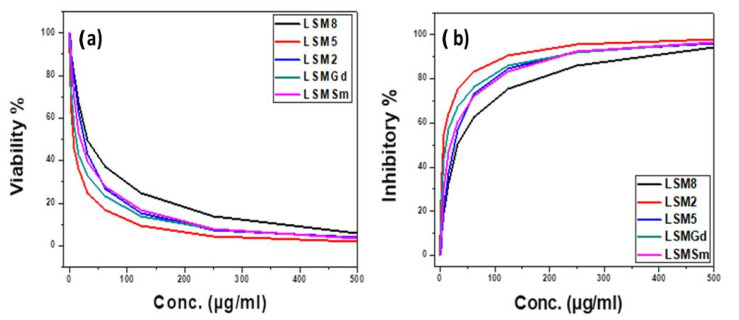
(**a**) The cell viability and (**b**) cell inhibitory (%) versus concentrations of LSM with different Sr^2+^ ion content and LSM with 0.2 Sm ions and 0.2 Gd ions at concentrations from 0 to 500 µg/m against HepG-2 cell line.

Figure 6 displays the SEM profiles of the HepG-2 cell line (H) control with untreated and treated samples in different concentrations from 10 to 500 µgm. Meanwhile, the cell viability and cell inhibition (%) versus concentrations of LSM with different Sr^2+^ ion content and LSM with 0.2 Sm ions and 0.2 Gd ions at concentrations from 0 to 500 µg/m against the HepG-2 cell line is indicated in Figure 7. Indeed, incubation of HepG2 cells with 0–500 μg/mL of LSM nanoparticles significantly decreased the cell viability. Anticancer effect outcomes indicate the outcrop of the essential cell death at higher concentrations of the samples. In this context, half maximal inhibitory concentration IC_50_ was also recorded for LSM samples tailored at various Sr^2+^ ions as well as doping with 0.2 Sm^3+^ or 0.2 Gd^3+^ ion ratios. For instance, IC_50_ for LSM8 was 31 ± 3.1 µg/mL. Moreover, IC_50_ was 6.73 ± 0.4, 25.8 ± 2.9, 11.4 ± 2.1, and 19.3 ± 3.8 µg/mL for LSM2, LSM5, LSMGd, and LSMSm, respectively. The presented values ascribed to IC_50_ for the LSM samples is lower than Ag-NPs at the concentration of 75 μg/mL [32], and nano CaO of 92.08 µg/mL [33] inhibits HepG-2 cell proliferation at about 50% (IC_50_) after 48 h of treatment. Besides, Priya et al. [34] demonstrated that the amount of biogenic silver nanoparticles synthesized using chitosan needed to decrease the cell viability of HepG2 cells to 50% of the initial population (IC_50_) was 48 ± 1.0 μg/mL, and the doxorubicin (standard) needed to reduce the viability of HepG2 cells to 50% of the initial population was 16 ± 1.0 μg/mL. The results can be discussed based on the particle size as well as the microstructures. Thereby, it is known that nanoparticle morphologies have a considerable impact on the cellular internalization. Sharp nanoparticle structures may introduce the membrane of endosome and localize to the cytoplasm [35,36]. Consequently, the cellular uptake of the spherical nanoparticles with different spherical ratios exhibited the bigger and faster absorption of the nanoparticles, which indicates that samples of LSM2 and LSMGd can be prospected as the favorite chemotherapeutic agents in liver hepatocellular carcinoma curing compared to other samples. 

### 3.5. Antimicrobial Study

All synthesized LSM samples were screened as antimicrobial against bacterial species, namely Staphylococcus aureus, Bacillussubtillus, Gram positive-like Proteus vulgaris, and Escherichia coli as Gram negative as well as against fungi species involved Aspergillus fiavus and Candida albicans. The activity of different concentrations of compounds is shown in Table 1. The results reveal that LSM with 0.2 and 0.8 Sr^2+^ ion concentrations were found to boost the activity towards Escherichiacoli (RCMB 010052) ATCC 25955 as Gram negative bacteria. On the other hand, LSM5, LSMGd, and LSMSm have activity towards Proteus vulgaris RCMB 004 (1) ATCC 13315.

### 3.6. Conclusions 

Lanthanum strontium manganite, La_1−x_Sr_x_MnO_3_, was developed, and distinguished in vitro studding for anticancer activities was predicted on intestinal carcinoma CACO-2 and human hepatocellular carcinoma cells HepG-2. The cell viability for CACO-2 and HepG-2 was decreased on LSM in a concentrated manner. The percentage of CACO-2 cell inhibition was found to reach 90 % with the addition of 500 µgm of the samples LSM2 and LSMGd. For half maximal inhibitory concentration, the IC_50_ of CACO-2 cell inhibition by LSM at a strontium content of 0.2 was 5.63 ± 0.42 µg/mL, and the value was increased with increased Sr^2+^ ion concentration by up to 0.8 to be = 25 ± 2.7 µg/mL. The IC_50_ of HepG-2 cell inhibition by LSM at a strontium content of 0.2 was 6.73 ± 0.4 µg/mL, and the value was increased with increased Sr^2+^ ion concentration by up to 0.8 to be = 31 ± 3.1 µg/mL. The addition of 0.2 of Gd^3+^ ion substituted LSM nanoparticles has a significant effect on CACO-2 cell and HepG-2 inhibition. Different LSM samples show activity towards Gram negative bacteria *Escherichia coli*, whereas LSM5, LSMGd, and LSMSm samples have a significant antimicrobial effect towards Gram negative bacteria *Proteus vulgaris*, and all samples possessed no detection towards fungi and Gram positive bacteria. LSM nanoparticles can be developed as possible chemotherapeutic agents in the remedy of intestinal carcinoma and liver hepatocellular carcinoma.

## Figures and Tables

**Table 1:** Activity of LSM samples at different conditions as antimicrobial agents.

	Sample Code	LSM8	LSM2	LSM5	Sm^3+^	Gd^3+^	Control
Tested Microorganisms							
Gram Negative Bacteria							Gentamycin
Escherichia coli(RCMB 010052)ATCC 25955		12	10	11	12	10	30
Proteus vulgaris RCMB 004 (1) ATCC 13315		NA	NA	12	13	10	25

## Data Availability

Not applicable.
